# Organelle specific fluorescent phenomics and transcriptomic profiling to evaluate cellular response to tris(1,3 dichloro 2 propyl)phosphate

**DOI:** 10.1038/s41598-022-08799-5

**Published:** 2022-03-18

**Authors:** Md Mamunul Haque, Taras Voitsitskyi, Jun-Seok Lee

**Affiliations:** 1 Department of Pharmacology, Korea University College of Medicine, Seoul, 02481 Republic of Korea; 2 Receptor.AI Inc., 16192 Coastal Highway, County of Sussex, Lewes, DE 19958 USA

**Keywords:** Chemical biology, Environmental sciences, Natural hazards

## Abstract

Tris(1,3-dichloro-2-propyl)phosphate (TDCPP) has been suspected to cause toxicity invertebrates, but its phenotypic effects and the underlying regulatory mechanism have not been fully revealed. Generally, cellular responses tightly control and affect various phenotypes. The scope of the whole organism or cellular toxicological phenotyping, however, has been limited, and quantitative analysis methods using phenotype data have not been fully established. Here, we demonstrated that fluorescence imaging of sub-organelle-based phenomic analysis together with transcriptomic profiling can enable a comprehensive understanding of correlations between molecular and phenomic events. To reveal the cellular response to TDCPP exposure, we obtained three sub-organelle images as fluorescent phenotypes. Transcriptomic perturbation data were measured from the RNA-seq experiment, and both profiling results were analyzed together. Interestingly, organelle phenomic data showed a unique fluorescent intensity increase in the endoplasmic reticulum (ER), and pathway analysis using transcriptomic data also revealed that ER was significantly enriched in gene ontology terms. Following the series of analyses, RNA-seq data also revealed potential carcinogenic effects of TDCPP. Our multi-dimensional profiling approach for organophosphate chemicals can uniquely correlate phenotypic changes with transcriptomic perturbations.

## Introduction

Organophosphate chemicals have been used as functional materials for various applications, including pesticides, plasticizers, and nerve gases^1^. Tris(1,3-dichloro-2-propyl)phosphate (TDCPP), an organophosphate, is widely used as a flame-retardant chemical. Since polybrominated diphenyl ethers (PBDEs) were identified as persistent organic pollutants and banned by the European Union in 2004, organophosphates have been used as primary substituents for PBDEs^2^. The annual production of TDCPP is estimated at around 22,000 metric tons, making it a high production chemical in the United States^3^, and it is commonly found in the fabrics used in caping, automobiles, and children’s foam products^4^. Because TDCPP is semi-volatile, it is continuously released from the products and its accumulation potentially affects the development of various diseases, such as non-alcoholic fatty liver disease, uterine fibroids, or hormonal disorders^3,5–8^. Recent studies have shown the adverse effects of TDCPP to increase the risk of cancer^9^, neurotoxicity, teratogenicity, and endocrine-disruptions^4,6,10–13^. For instance, TDCPP exposed zebrafish showed developmental abnormalities^12^, disrupting sex-hormone levels^11^, and dysregulated thyroid hormone (TH)-responsive genes^13^. TDCPP also showed neurotoxicity in cultured neuroendocrine cells^4^, leg and wing weakness in chickens^14^, and reduced thyroxine levels in humans, chicken embryos, and zebrafish^6,13^. Because TDCPP has been classified as a Cat I* compound from the Quick Chemical Assessment Tool^15^, the majority of studies have focused on the risk assessment of TDCPP or animal toxicology studies; however, detailed phenotypic changes or molecular mechanisms of toxic effects with high precision of sub-cellular organelle levels have not yet been explored.

Although phenotype profiling provides information on systemic toxicity, quantitative analysis for such profiling is not always straightforward^16^. Our group has been developing fluorescent imaging agents and image-based phenomics analysis at the single-cell level. In a previous study, we demonstrated that a chemical sensor for superoxide radical anions could visualize influenza virus infections at the individual cell level and that the fluorescent pattern of host cells could distinguish subtypes of the influenza virus^17^. As demonstrated in a previous study, fluorescent phenotype images provide high content information, such as cell morphology, mitochondrial membrane potential, or superoxide level, depending on the properties of fluorescent probes^18,19^. In this study, we examined the cellular phenotype of subcellular organelles upon TDCPP exposure and analyzed the phenotypic changes together with transcriptomic perturbations to understand the molecular mechanism of TDCPP toxicity.

## Materials and methods

### Chemicals

TDCPP was purchased from Sigma (Sigma-Aldrich, #32951, Oakville, ON), and stock solutions were prepared in dimethyl sulfoxide (DMSO) (Sigma-Aldrich, #34869, Oakville, ON) to yield a concentration of 100 mM.

### Cytotoxicity assay

HeLa cells (obtained from Korean Cell Line Bank, KCLB#10002) were grown in DMEM supplemented with 10% FBS, 100 units/mL penicillin, and 100 μg/mL streptomycin in a humidified incubator containing 5% CO_2_ at 37 °C. For the cytotoxicity assay, HeLa cells were seeded in a 96-well plate at a density of 1 × 10^4^ cells/well and incubated for 24 h. Cells were treated with various concentrations (0–500 µM) of TDCPP for 4 and 24 h. After incubation, 10 µL of EZ-CyTox reagent was added to the cells for 30 min as manufacture’s standard protocol, and the absorbance was measured at 450 nm using a FlexStation 3 spectrophotometer (Molecular Devices, Sunnyvale, CA, USA). The mean absorbance value of three replicate wells was analyzed with non-treated cells as control (Fig. S1).

### Sub-cellular organelle phenomics profiling

To check the effects of the compounds on different organelles, three different organelle trackers were treated for 30 min (ER-Red BODIPY glibenclamide, 1 µM, Mito-Green FM, 1 µM, and Hoechst, 1 mg/Ml). The cells were then washed twice with fresh DMEM medium and imaged under three fluorescence channels (blue; λ_ex_ = 350/50 nm and λ_em_ = 460/50 nm, green; λ_ex_ = 480/40 nm and λ_em_ = 527/30 nm, and red; λ_ex_ = 546/10 nm and λ_em_ = 585/40 nm) using a Leica DMi8 microscope (Leica, Germany) at 20× objectives. Image analysis results using two modules (AreaShape_Perimeter and Intensity_MeanIntensity) from CellProfiler software are plotted in this manuscript. Values of parameters from individual cells in an experimental well were averaged, and the mean value of six replicate wells was used for bar graph plotting and further statistical analyses.

### Transcriptomics analysis of RNA-seq

HeLa cells were treated with different concentrations of TDCPP (up to 500 µM) for 2 and 24 h. Total RNA was isolated using TRIzol reagent (Invitrogen, Carlsbad, CA, USA). Then, the RNA-seq experiment was performed using a commercially available Illumina HiSeq 4000 sequencer (Macrogen Inc., Korea). Gene expression levels were normalized as reads per kilobase of transcript per million reads (RPKM). FastQC v0.11.7 was used for RNA-seq data processing to examine the average base quality per cycle. Acceptable criteria were set as a phred quality score of ≥ 20 (99%). Before analysis, the adapter sequence was removed using the Trimmomatic 0.38 program and low-quality bases were also removed. Pre-processed trimmed reads were mapped to the reference genome (Genome_build: UCSC hg19), and transcript assembly was performed using StringTie (v1.3.4d) to generate expression profile values for each sample. More detailed processing procedures and full expression data are available in the NCBI GEO database (accession #: GSE147560). Differentially expressed genes (DEGs) were determined by following the threshold of fold change > 2.0, and p-value < 0.05. Additionally, Ingenuity Pathway Analysis (IPA) was used for pathway mapping.

### Quantitative reverse-transcription polymerase chain reaction (qRT-PCR)

Total RNA was isolated using TRIzol, and the concentration and purity of RNA were measured using Nanodrop (Thermo Fisher, MA, USA). cDNA synthesis was performed using a QuantiTect Reverse Transcription Kit (QIAGEN, #205313, MA, Germany). The primers were synthesized by Macrogen Inc. (Korea), and qRT-PCR was performed using SsoFast™ EvaGreen® Supermix (BIO-RAD, #1725201, MA, USA) according to manufacturer instructions. A melting curve was used to check the purity and specificity of the PCR products in each assay. Thermal cycling was set at 95 °C for 10 min, followed by 40 cycles of 95 °C for 15 s and 58 °C for 1 min. The mRNA expression levels were expressed as fold change, and ACTB mRNA was used as a reference gene. qRT-PCR analyses were performed in triplicate for the tested genes.

### Data preparation scheme

In order not to lose potentially valuable information inside different treatment conditions, we used the fold change of each treatment condition compared to the control sample (Control vs Treatment; Fig. S2, green boxes) and. also calculated fold change values for comparing the time-dependent (24 h vs. 4 h; Fig. S2, blue boxes) and concentration-dependent groups (4 µM vs. 20 µM vs. 100 µM; Fig. S2, pink boxes).

### Functional pathway analysis

Gene Ontology (GO Enrichment Analysis, http://geneontology.org/) was used to perform general functional pathway analysis. Cellular components, molecular functions, and biological processes were examined for enrichment. A false discovery rate (FDR) adjusted p-value < 0.05, was considered to be significantly altered. For the analysis, we chose significantly up-/downregulated (absolute fold change ≥ 2) genes in at least one comparison pair (time-dependent group and/or concentration-dependent group). The Python 3.6, scikit-learn library method was used for visualization. Raw signal normalized counts from RNA-seq data (calculated as transformed logarithm and normalization using the quantile method) were used for algorithm processing.

The Ingenuity^®^ Pathway Analysis (IPA) tool was used as a pathway topology tool to identify the potential impact of TDCPP on canonical signaling pathways, biological functions, disease progression, toxic effects, and molecular networks. Upstream regulators and causal networks predicted to participate in HeLa cell metabolism disruption were additionally revealed. The fold change threshold in IPA was −1.5 ≥ fold change value ≥ 1.5, because filtering with absolute fold change ≥ 2 resulted in limited analysis.

## Results

### Fluorescent sub-organelle phenomic profiling

To visualize the phenotypic changes from individual cells at the sub-organelle level, we chose organelle-selective imaging fluorophores. Three organelles (the nucleus, mitochondria, and endoplasmic reticulum (ER)) were chosen because these organelles cover the major structural components of cells. Each organelle image was obtained using distinct color fluorophores; nuclei were stained with Hoechst, mitochondria were stained with MitoTracker™ Green FM, and ER was imaged using ER-Red BODIPY glibenclamide. We treated the cells with three fluorophores immediately before obtaining cell images. Cells were pre-incubated with TDCPP at five serial concentration points with six replicates of nine image sets per experiment (9 images/exp × 5 concentration points × 6 replicates × 3 organelle trackers). A total of 810 images were analyzed using CellProfiler, an image-based high-content screening (HCS) analysis program^20^.

**Figure 1:**
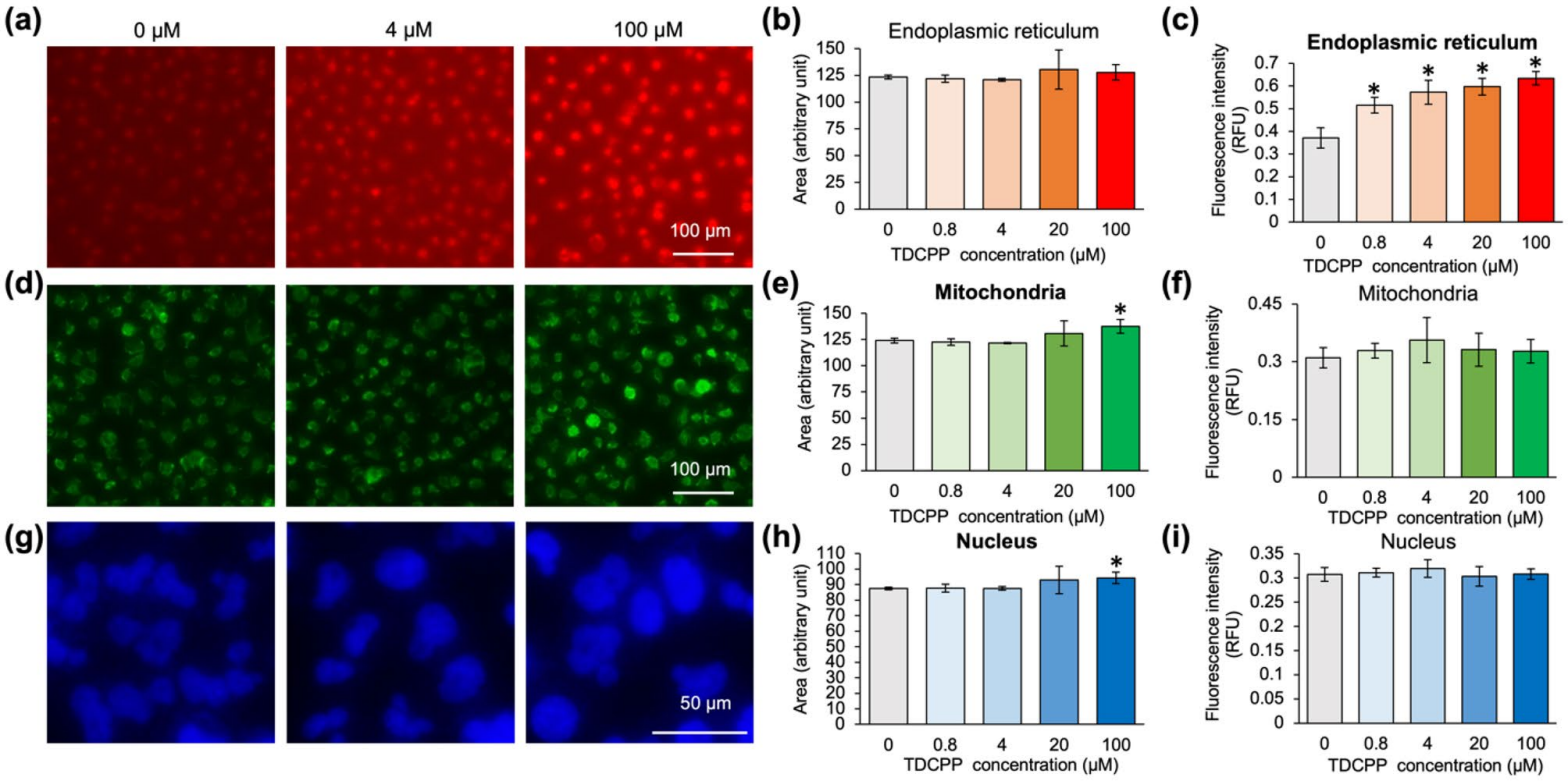
Fluorescence phenomic signature of TDCPP treated Hela cells. **(a)** Fluorescence live-cell images of ER, statistical values of **(b)** stained area from fluorescent ER images, **(c)** fluorescent intensity of ER, **(d)** fluorescence live-cell images of mitochondria, statistic values of **(e)** stained area from fluorescent mitochondria images, **(f)** fluorescent intensity of mitochondria, **(g)** fluorescent live-cell images of a nucleus, statistic values of **(h)** stained area from fluorescent nucleus images, **(i)** fluorescent intensity of nucleus. TDCPP was pre-incubated with Hela cells for 24 h and organelle trackers were treated before obtaining images. Data are represented as mean ± SEM (n = 6 well, *p < 0.001).

From the fluorescence phenomic analysis, we analyzed the organelle size and fluorescence intensity of each organelle tracker. As summarized in Fig. 1, we observed a unique fluorescence increment from ER images (1.7-fold increase), despite negligible changes in the stained area representing organelle size. A subtle increase in nucleus size was observed but the intensity did not change. Unlike the ER or nucleus, mitochondria did not exhibit apparent size or staining intensity perturbations. These results suggest that TDCPP induced homeostatic conditional changes to the ER because the ER tracker senses local environmental condition changes at the ER.

Notably, the sub-organellar phenomic signature reported much more apparent phenotypic changes than conventional whole-cell level cytotoxicity profiling. Based on the cytotoxicity assay using 0 to 500 µM TDCPP treatment conditions, all concentration points showed more than 80% cell viability after 24 h incubation in HeLa cells (Fig. S1). Conventional cytotoxicity assays provide limited information about compound toxicity. In contrast, the fluorescent sub-organelle phenomic profile has merit in that it provides spatial-selective responses at specific sub-organelles as well as multi-dimensional information about organelle size, shape, and fluorescent intensity that potentially represent molecular environmental changes.

Because TDCPP-treated cells showed unique fluorescence responses in the ER, we suspected that these phenomena might be originated from ER stress signaling pathways. It was previously reported that ER stress alters the sizes and staining intensities of ER^21,22^. To further investigate the individual transcriptomic perturbation profile of TDCPP, we next conducted a global transcriptomic profiling analysis.

### Transcriptomic profiles

**Figure 2:**
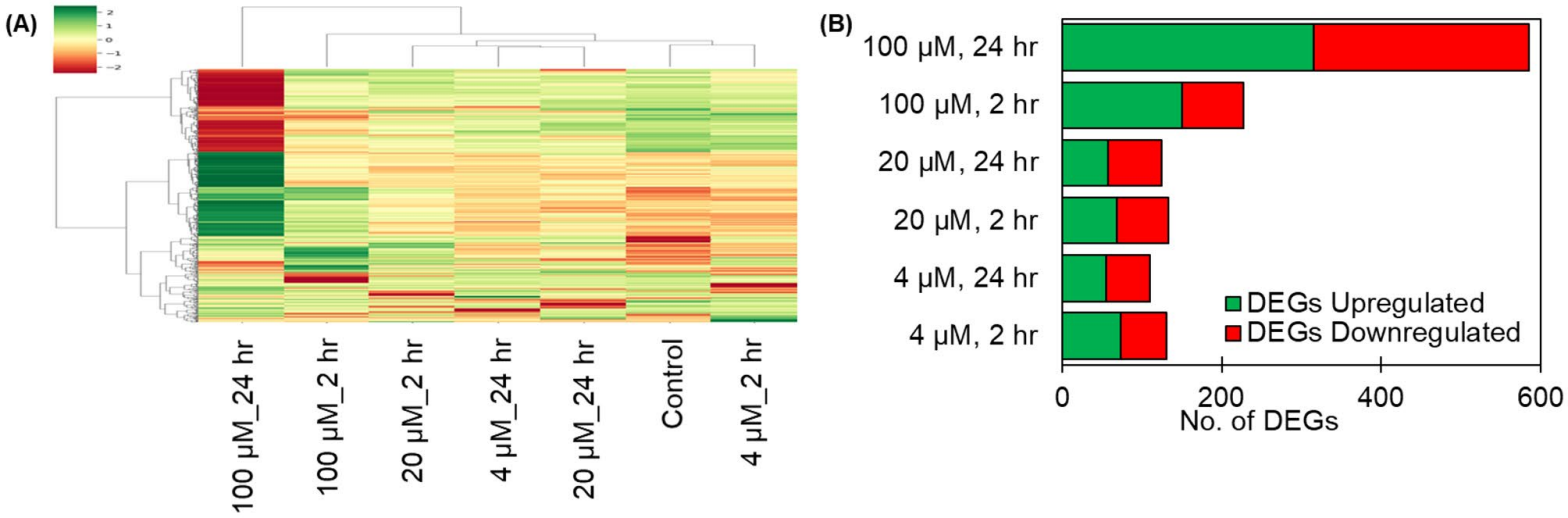
Differentially expressed genes in RNA-seq experiment. **(A)** Unsupervised hierarchical clustering heatmap of DEG expression based on z-score data of RNA-seq transcriptome. Each column represents an experimental condition and each row represents a single gene. Green means upregulation and red means downregulation. The vertical distances on each indicate the degrees of similarity between genes and the horizontal distances on each column indicate the degrees of similarity between the experimental groups. **(B)** Number of DEGs in each comparison condition. The vertical axis corresponds to the experimental comparison groups, and the horizontal axis displays the number of DEGs.

To reveal global transcriptomic perturbation signatures, RNA-seq experiments were conducted on HeLa cells after short (2 h) and prolonged (24 h) treatment of TDCPP (experimental schemes in Fig. S2). Initially, a hierarchical agglomerative clustering algorithm was applied to identify differentially expressed genes (DEGs) with the same mode of change under treatment conditions (Fig. 2A).

Analysis was performed on the genes that were significantly changed in at least one condition. Several noticeable expressional alterations occurred at 100 µM TDCPP (586 DEGs for 24 h incubations, and 227 DEGs for 2 h incubations). The corresponding numbers for the remaining four sets (low and medium concentrations of TDCPP) did not exceed 133 DEGs. Overall, 1312 DEGs were detected in the TDCPP-treated versus vehicle group (Fig. 2B). Among these genes, 719 genes were upregulated and 593 genes were downregulated, indicating that TDCPP induces more up-regulation in cellular responses.

**Figure 3:**
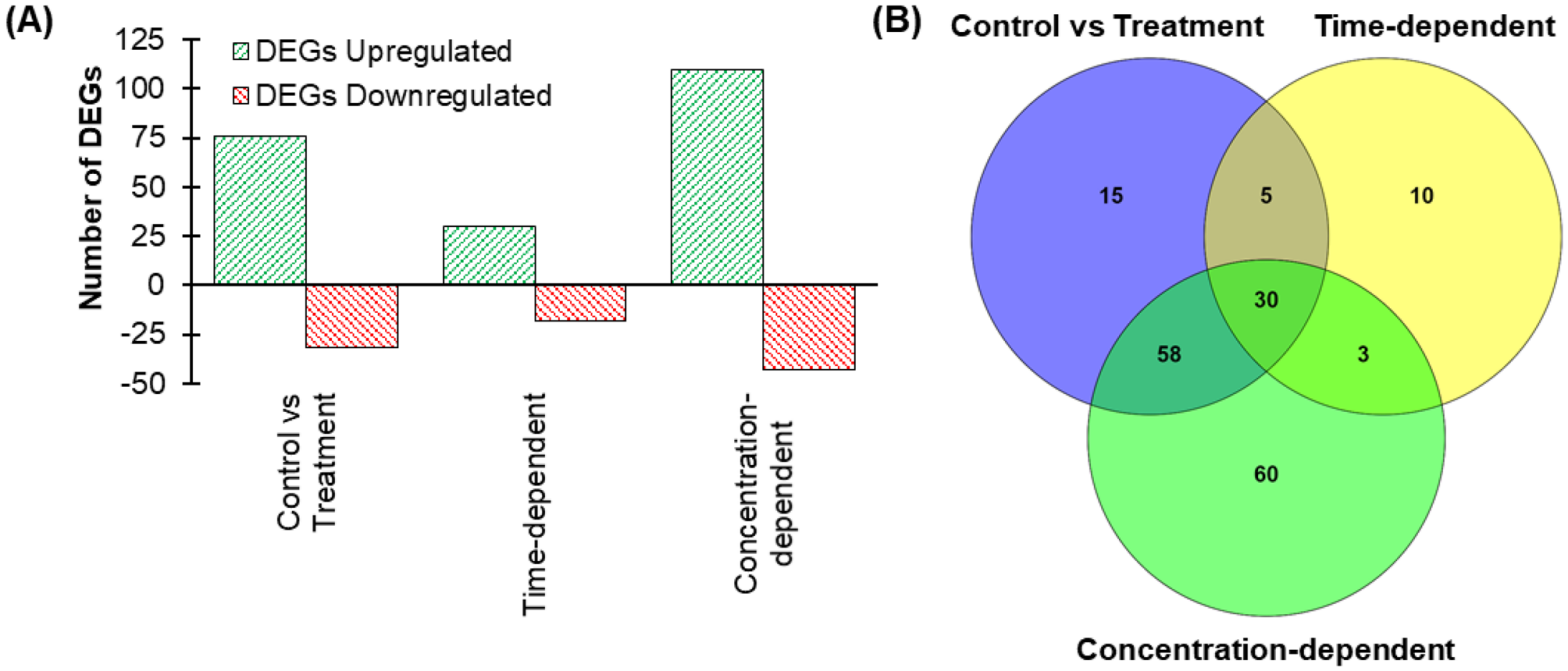
**(A)** The significantly altered genes in the RNA-seq experiment based on the absolute Z-score ≥ 2 (threshold corrected p-value < 0.05). **(B)** Venn diagram showing the number of genes and their co-existence in different experimental conditions.

From the above list, we further narrowed down significantly perturbed genes by applying more stringent criteria as significantly altered targets, which are absolute Z-scores higher than 2 (threshold corrected p-value < 0.05). The genes of these groups were separated; the corresponding full genes are listed in Table S1. Based on these criteria, 108 genes were altered in the “Control vs Treatment group,” 48 genes in the “Time-dependent group” and 151 genes in the “Concentration-dependent group” (Fig. 3). Among the 181 genes, 30 genes were common in all groups.

Gene ontology pathway analysis (GO analysis) was performed using these 181 DEGs. The dysregulated genes were mainly associated with ER (47 genes), apoptotic processes (41 genes), extracellular exosomes (38 genes), and protein metabolism (20 genes). The individual gene information is listed in Table S2. The most enriched terms in the “control vs. treatment group” were predominantly related to exosomes, endoplasmic reticulum, protein metabolism, and apoptosis. Particularly, endoplasmic reticulum and apoptosis-related DEGs were also altered in.

“Time-dependent group,” whereas secretion-disruption-related DEGs were altered in the “Concentration-dependent group.” Overall, GO analysis revealed that ER, apoptosis, and extracellular exosomes were significantly altered in TDCPP-treated HeLa cells. Notably, DEG analysis also showed ER as the most significantly associated GO term, which is consistent with the fluorescence phenotypic profiling.

### Pathway analysis

To further investigate cellular responses, we examined the pathway analysis. The list of altered pathways was analyzed using IPA tools and hit pathways were filtered using the following conditions: (i) −log(*p*-value) ≥ 1.3 and (ii) absolute Z-score ≥ 2 in “Control vs Treatment group.” From the pathway analysis, we discovered that neuroinflammation signaling, sirtuin signaling, and the apelin endothelial signaling pathway were the major disturbing pathways (Fig. S3). Exposure to low concentrations of TDCPP at 4 µM particularly affected the apelin endothelial and neuroinflammation signaling pathways. Additionally, prolonged exposure to high concentrations of TDCPP (100 µM) affected a series of cholesterol biosynthesis pathways. This showed that continuous TDCPP exposure potentially perturbed much broader signaling pathways. To better understand and identify the cascade of signaling pathways, we searched upstream transcriptional regulating factors leading to the gene expression signatures that we obtained using upstream regulator analysis.

**Figure 4:**
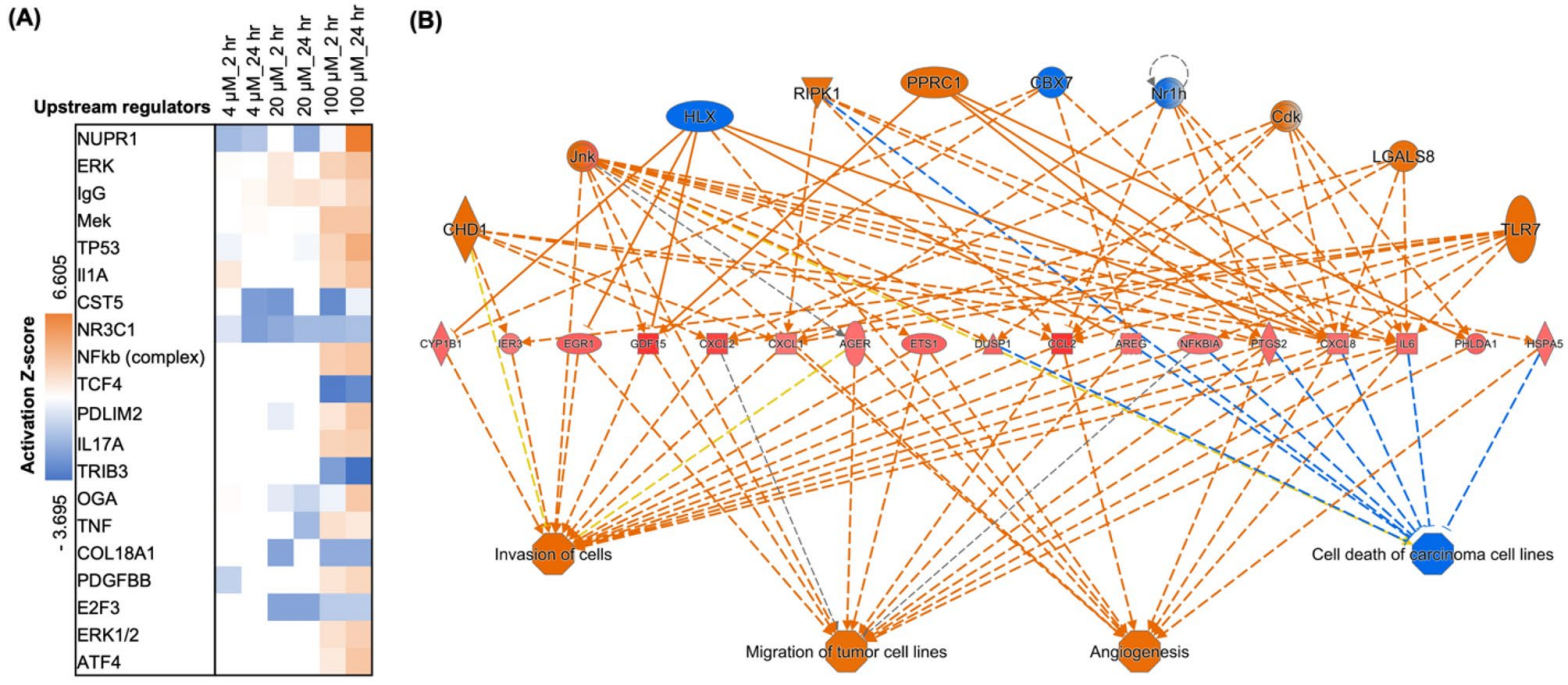
IPA upstream regulators and functions based on RNA-seq of TDCPP-treated HeLa cells with absolute Z-score ≥ 2 at least in one condition and −log(p-value) ≥ 1.3. **(A)** Top 20 upstream regulators in the group Control vs. Treatment. Results based on fold change values calculated by comparing gene expression in 6 treatment conditions to control. Positive Z-score indicates activation, and a negative score indicates inhibition. **(B)** Regulator effects of 2 most consistent networks based on RNA-seq of TDCPP treated HeLa cells. Both networks were constructed using data from 100 µM, 2 h/control (consistency score equal to 34.9).

As expected, the most significant regulators were discovered in the group treated with 100 µM TDCPP (Fig. 4A). The most activated regulator was *NUPR1*, followed by *TP53*. In contrast, the most inhibited regulator was *TRIB3*, followed by *TCF4*. Some notable regulators working at low concentrations were *CST5* and *NR3C1*. *IgG* and *E2F3* also played roles in the middle-concentration range. Next, we proceeded with regulator effect analysis, in which the algorithm connects upstream regulators, diseases, and functions into a merged network. Regulator effect analysis revealed angiogenesis and invasion of the tumor cell line (Fig. 4B, Fig. S4). This observation implies that exposure to TDCPP eventually increases the risk of cancer by affecting blood vessel generation and metastasis, which is also consistent with previous studies^23^.

Among these transcription regulators, activated *NUPR1* (nuclear protein 1) is highly associated with primary tumors and metastases, including breast, thyroid, and pancreatic cancer^24,25^. Interestingly, recent studies of organophosphates also revealed their associations with thyroid and pancreatic cancers^26,27^. TDCPP dramatically reduces plasma thyroxine (T4) and triiodothyronine (T3) with TDCPP exposure in females^28^, and both T3 and T4 play roles in tumor-promoting effects in hormonally related cancers such as pancreatic and prostate cancer^27^. Besides, the negative regulator *TCF4* is known for tumor invasiveness^29^ and is also an important factor in persistent proliferation in case of loss of its expression^30,31^. Although there are few studies on the details of TDCPP exposure and *TCF4* expression, there are notable functional connections to cancer progression and its downstream networks. Thus, our results highlight the importance of upstream regulator analysis to reveal the major triggering target of biological events for long-term exposure to substances.

### qRT-PCR validation of early responding genes

Although the majority of DEGs showed temporal responses at certain concentrations or incubation points, some genes surprisingly exhibited consistent expression patterns. These genes may be considered early responding genes upon TDCPP exposure and can be potentially utilized as markers for monitoring assays. From the series of different treatment groups (at least in four groups), we identified five genes (*LIF, TXNDC5, NDUFA5, RLIM,* and *SERF1A*). To validate such consistent expression signatures, we further conducted a qRT-PCR analysis of the selected genes.

In brief, HeLa cells were treated with 0, 4, 20, and 100 µM for 4 h and 1 day. Then, the cells were harvested and obtained data using qRT-PCR. As expected, qRT-PCR data showed early up-regulation of *LIF* and downregulation of *TXNDC5, NDUFA5, RLIM,* and *SERF1A* (Fig. S5). *LIF* gene encodes interleukin 6 (IL-6) class cytokine that produces the acute phase proteins, such as opsonins, that stimulate pro-inflammatory immune responses. Recent studies have proved the connections between IL-6 and ER stress^32^, or ER phenotype changes^33^. In addition, thioredoxin domain containing 5 (encoded by *TXNDC5*) and NADH-ubiquinone oxidoreductase (encoded by *NDUFA5*) are localized in ER. It is also noteworthy that ring finger protein (encoded by *RLIM*) and small EDRK-rich factor (encoded by *SERF1*) are associated with special interactions with ER-localized proteins, UBX domain protein (encoded *UBXN1*), and ELOVL fatty acid elongase-7 (encoded by *ELOVL7*), respectively^34,35^.

These finding strongly suggests our unique ER fluorescent phenotype data represent a series of underlying genomic regulations. With the support from the above, our simple but quantitative fluorescent phenotyping methods visualized cellular responses upon exposure to TDCPP, and successfully demonstrated its unique merits for evaluation of chemical toxicity. Together with the whole-genome-wide transcriptome analysis, it is possible to expand our understanding of the molecular action point from the genome to the sub-organelle level. We expect such image-based multi-dimensional profiling will open up new windows to evaluate and comprehend the mode of action of organophosphate chemicals.

## Conclusion & discussion

In this study, we conducted a parallel measurement of fluorescence phenomic alterations and transcriptomic profiles upon treatment of TDCPP in HeLa cells. We found a dramatic fluorescence increase in ER using phenomic data, and ER was also significantly enriched in gene ontology on pathway analysis using transcriptomic data. These observations indicate that ER-related pathways were perturbed by TDCPP. Our study demonstrated the potential of combination analysis of fluorescence sub-organelle phenomics and transcriptomics. However, there are still shortcomings including relatively small sample size (three sub-organelles and one flame retardant). Further extension of this study with additional sub-organelles is still required to fully evaluate TDCPP-induced phenomic damages. Overall, our finding suggests multi-dimensional profiling approaches to evaluate the toxicity of organophosphate chemicals like TDCPP by correlating phenotypic changes with transcription perturbations.

## Supplementary Information

Supplementary Figures.

Supplementary Tables.

## References

[1] Terry AV (2012). Functional consequences of repeated organophosphate exposure: Potential non-cholinergic mechanisms. Pharmacol. Ther..

[2] Jain T, Jamali D (2016). Looking inside the black box: The effect of corporate governance on corporate social responsibility. Corp. Gov. Int. Rev..

[3] van der Veen I, de Boer J (2012). Phosphorus flame retardants: Properties, production, environmental occurrence, toxicity and analysis. Chemosphere.

[4] Dishaw LV (2011). Is the PentaBDE replacement, tris (1,3-dichloro-2-propyl) phosphate (TDCPP), a developmental neurotoxicant? Studies in PC12 cells. Toxicol. Appl. Pharmacol..

[5] Carignan CC (2013). Predictors of tris(1,3-dichloro-2-propyl) phosphate metabolite in the urine of office workers. Environ. Int..

[6] Meeker JD, Stapleton HM (2010). House dust concentrations of organophosphate flame retardants in relation to hormone levels and semen quality parameters. Environ. Health Perspect..

[7] Lee G (2020). Exposure to organophosphate esters, phthalates, and alternative plasticizers in association with uterine fibroids. Environ. Res..

[8] Negi CK, Bajard L, Kohoutek J, Blaha L (2021). An adverse outcome pathway based in vitro characterization of novel flame retardants-induced hepatic steatosis. Environ. Pollut..

[9] Andresen JA, Grundmann A, Bester K (2004). Organophosphorus flame retardants and plasticisers in surface waters. Sci. Total Environ..

[10] Farhat A (2013). In Ovo effects of two organophosphate flame retardants-TCPP and TDCPP-on pipping success, development, mRNA expression, and thyroid hormone levels in chicken embryos. Toxicol. Sci..

[11] Liu X, Ji K, Choi K (2012). Endocrine disruption potentials of organophosphate flame retardants and related mechanisms in H295R and MVLN cell lines and in zebrafish. Aquat. Toxicol..

[12] McGee SP, Cooper EM, Stapleton HM, Volz DC (2012). Early zebrafish embryogenesis is susceptible to developmental TDCPP exposure. Environ. Health Perspect..

[13] Wang Q (2013). Exposure of zebrafish embryos/larvae to TDCPP alters concentrations of thyroid hormones and transcriptions of genes involved in the hypothalamic-pituitary-thyroid axis. Aquat. Toxicol..

[14] Ulsamer AG, Osterberg RE, McLaughlin J (1980). Flame-retardant chemicals in textiles. Clin. Toxicol..

[15] Bajard, L., Melymuk, L. & Blaha, L. Prioritization of hazards of novel flame retardants using the mechanistic toxicology information from ToxCast and Adverse Outcome Pathways. *Environ. Sci. Eur*. 10.1186/s12302-019-0195-z (2019).

[16] Houle D, Govindaraju DR, Omholt S (2010). Phenomics: The next challenge. Nat. Rev. Genet..

[17] Hong SC (2018). Discrimination of Avian Influenza Virus subtypes using host-cell infection fingerprinting by a sulfinate-based fluorescence superoxide probe. Angew. Chem. Int. Ed. Engl..

[18] Massey AJ (2018). A high content, high throughput cellular thermal stability assay for measuring drug-target engagement in living cells. PLoS ONE.

[19] Lee JS, Lee JW, Kang N, Ha HH, Chang YT (2015). Diversity-oriented approach for chemical biology. Chem. Rec..

[20] Kamentsky L (2011). Improved structure, function and compatibility for Cell Profiler: Modular high-throughput image analysis software. Bioinformatics.

[21] Sicari D, Delaunay-Moisan A, Combettes L, Chevet E, Igbaria A (2020). A guide to assessing endoplasmic reticulum homeostasis and stress in mammalian systems. FEBS J..

[22] Schuck S, Prinz WA, Thorn KS, Voss C, Walter P (2009). Membrane expansion alleviates endoplasmic reticulum stress independently of the unfolded protein response. J. Cell Biol..

[23] Betts KS (2013). Exposure to TDCPP appears widespread. Environ. Health Perspect..

[24] Santofimia-Castano, P. *et al.* Targeting the stress-induced protein NUPR1 to treat pancreatic adenocarcinoma. *Cells*. 10.3390/cells8111453 (2019). 10.3390/cells8111453 PMC6912534 31744261

[25] Santofimia-Castano P (2019). Ligand-based design identifies a potent NUPR1 inhibitor exerting anticancer activity via necroptosis. J. Clin. Invest..

[26] Zhang X (2021). Genetic comprehension of organophosphate flame retardants, an emerging threat to prostate cancer. Ecotoxicol. Environ. Saf..

[27] Moeller LC, Fuhrer D (2013). Thyroid hormone, thyroid hormone receptors, and cancer: A clinical perspective. Endocr. Relat. Cancer.

[28] Deziel NC (2018). A case-control study of exposure to organophosphate flame retardants and risk of thyroid cancer in women. BMC Cancer.

[29] Sanchez-Tillo E (2011). Beta-catenin/TCF4 complex induces the epithelial-to-mesenchymal transition (EMT)-activator ZEB1 to regulate tumor invasiveness. Proc. Natl. Acad. Sci. U. S. A..

[30] van de Wetering M (2002). The beta-catenin/TCF-4 complex imposes a crypt progenitor phenotype on colorectal cancer cells. Cell.

[31] Shang S, Hua F, Hu ZW (2017). The regulation of beta-catenin activity and function in cancer: Therapeutic opportunities. Oncotarget.

[32] Sanchez CL, Sims SG, Nowery JD, Meares GP (2019). Endoplasmic reticulum stress differentially modulates the IL-6 family of cytokines in murine astrocytes and macrophages. Sci. Rep..

[33] Ayaub EA (2019). IL-6 mediates ER expansion during hyperpolarization of alternatively activated macrophages. Immunol. Cell Biol..

[34] Alexandru G (2008). UBXD7 binds multiple ubiquitin ligases and implicates p97 in HIF1alpha turnover. Cell.

[35] Luck K (2020). A reference map of the human binary protein interactome. Nature.

