# Supplementary material for: Organelle specific fluorescent phenomics and transcriptomic profiling to evaluate cellular response to tris(1,3 dichloro 2 propyl)phosphate
Source: Sci Rep. 2022 Mar 18;12:4660. doi: 10.1038/s41598-022-08799-5 (PMC8933422; doi:10.1038/s41598-022-08799-5)
Supplement: Supplementary file 1 — Supplementary Figures. [file 41598_2022_8799_MOESM1_ESM.docx]

Supporting Information

**Organelle specific Fluorescent Phenomics and Transcriptomic Profiling to Evaluate Cellular Response to Tris(1,3 dichloro 2 propyl)phosphate**

Md Mamunul Haque^†,¥^, Taras Voitsitskyi^†,¥^, Jun-Seok Lee^†,*^

^†^Department of Pharmacology, Korea University College of Medicine, Seoul 02481, Korea

¥These authors contributed equally.

**Figure S1.** Cellular toxicity effect of TDCPP. Cellular viability of HeLa cells was determined after 4 hr and 24 hr of incubation with different concentration. Each concentration was examined as triplicates and mean, and standard deviation are plotted.

**Figure S2.** Experimental scheme of transcriptome profiling using RNA-SEQ

**
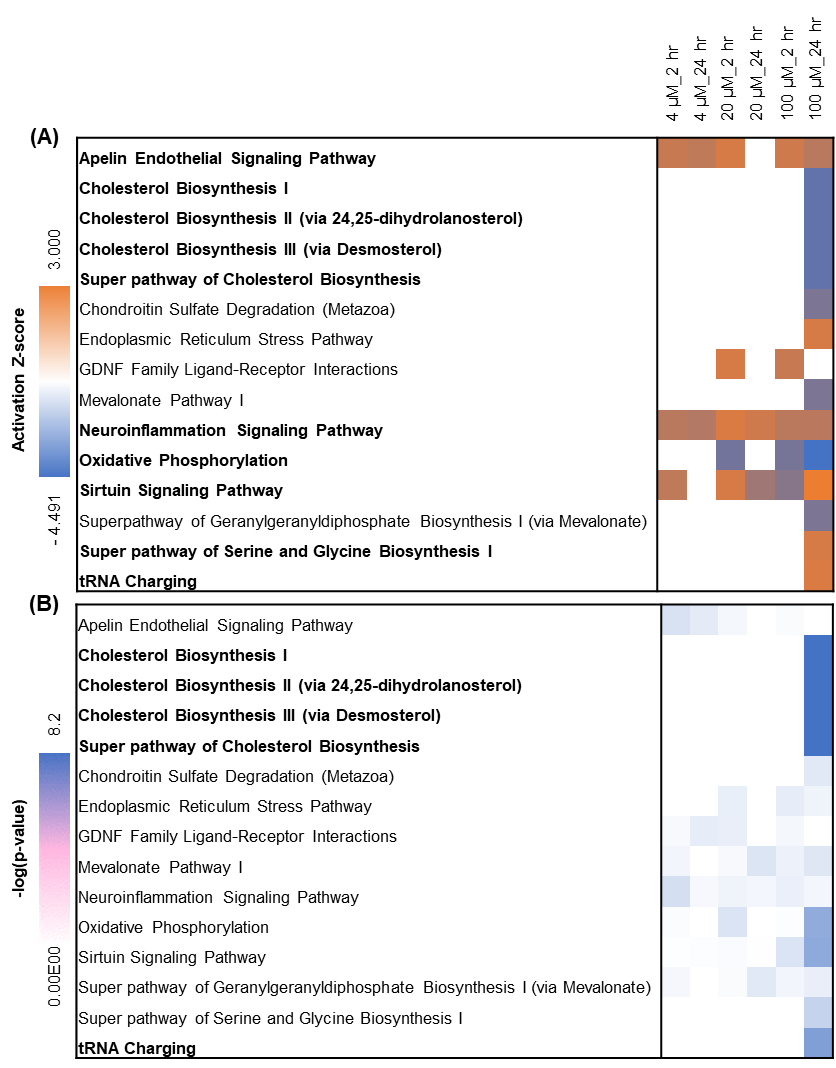
**

**Figure S3.** Canonical pathway analysis of TDCPP treated samples. Results based on fold change values calculated by comparing genes expression in 6 treatment conditions to control. **(A)** Positive Z-score means activation and negative score indicates inhibition. **(B)** Corresponding -log(p-values) for each pathway and comparison condition.


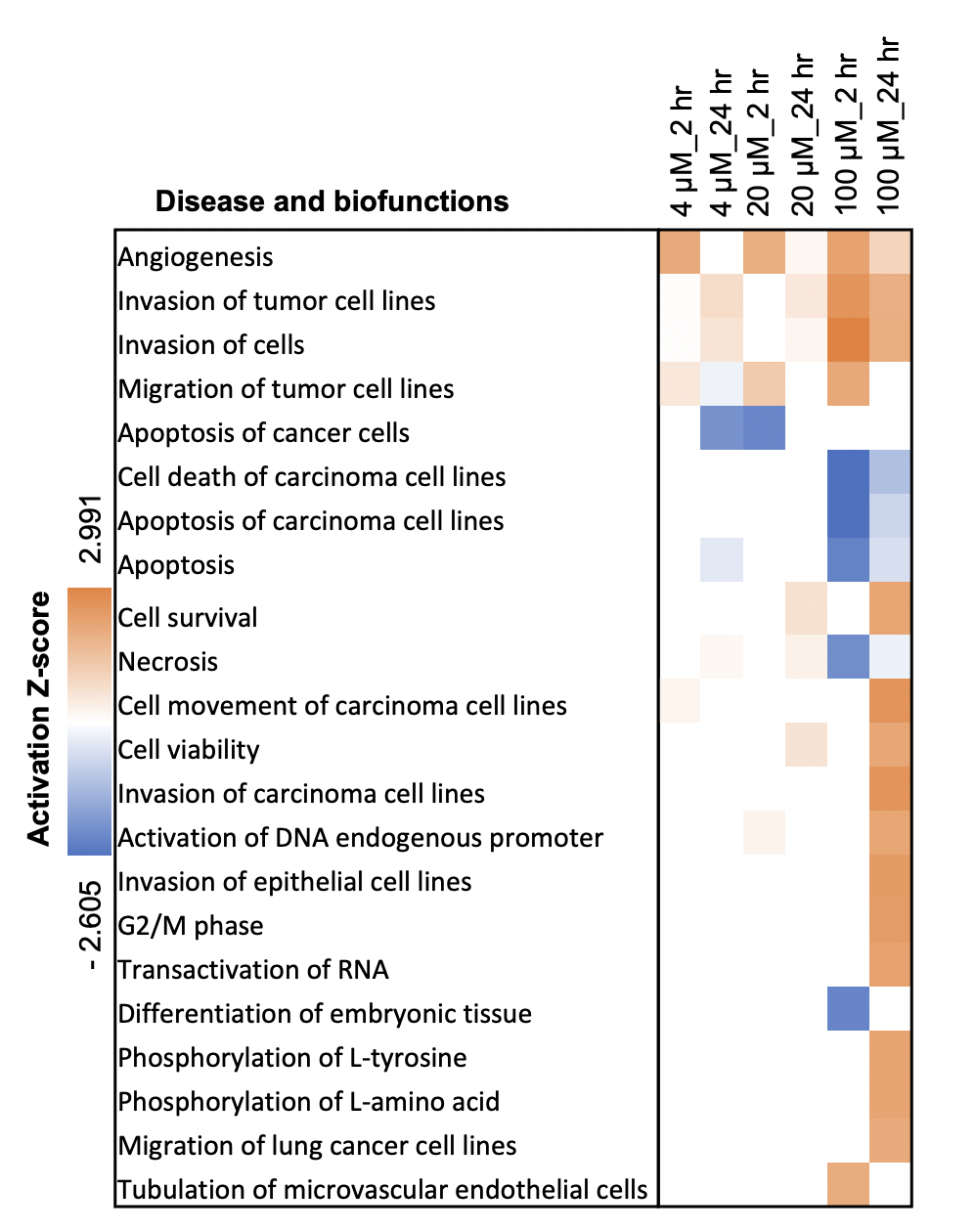


**Figure S4.** Diseases and biofunctions from IPA upstream regulators analysis. Control (NT) vs Treatment group analysis (absolute Z-score ≥ 2 at least in one condition and -log(p-value) ≥ 1.3).


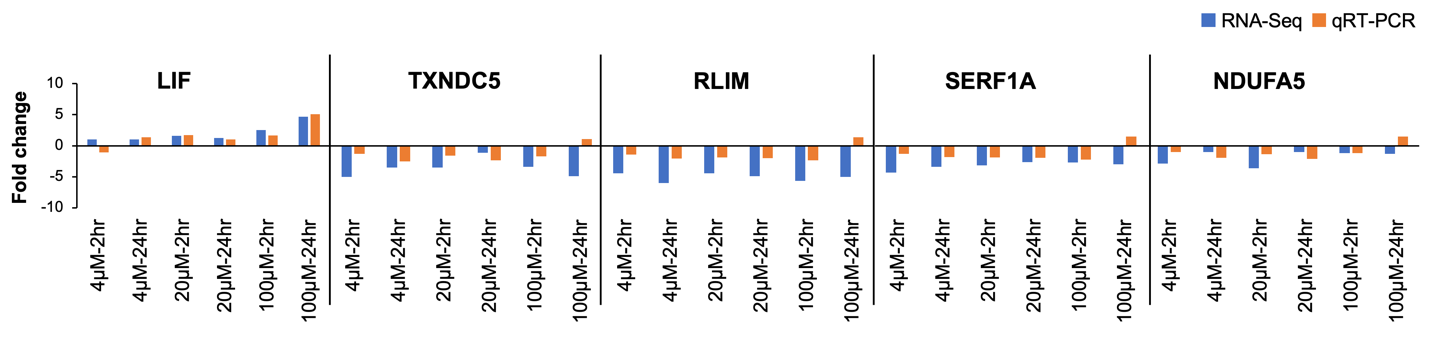


**Figure S5**. qRT-PCR of early responding genes. HeLa cells were treated with different concentrations of TDCPP for 2 time-points. Then, cell was harvested, and qRT-PCR was performed for 6 most consistently expressed genes.
